# Supplementary material for: A Critical View of the Application of the APEX Software (Aqueous Photochemistry of Environmentally-Occurring Xenobiotics) to Predict Photoreaction Kinetics in Surface Freshwaters
Source: Molecules. 2019 Dec 18;25(1):9. doi: 10.3390/molecules25010009 (PMC7017383; doi:10.3390/molecules25010009)
Supplement: Supplementary file 1 [file molecules-25-00009-s001.zip › 3.2.4_gcc-4.4.0.zip!/3.2.4_gcc-4.4.0/3.2.4_gcc-4.4.0/doc/octave/html/liboctave/Bounds.html]

Bounds - Octave C++ Classes


Next: Linear Constraints,
Previous: Objective Functions,
Up: Optimization


---

### 9.2 Bounds

— : **Bounds** (void)  
— : **Bounds** (int n)  
— : **Bounds** (const ColumnVector lb, const ColumnVector ub)  
— : **Bounds** (const Bounds &a)

— : Bounds& **operator =** (const Bounds &a)

— : Bounds& **resize** (int n)

— : double **lower\_bound** (int index) const;  
— : double **upper\_bound** (int index) const;

— : ColumnVector **lower\_bounds** (void) const;  
— : ColumnVector **upper\_bounds** (void) const;

— : int **size** (void) const;

— : Bounds& **set\_bound** (int index, double low, double high)

— : Bounds& **set\_bounds** (double low, double high)  
— : Bounds& **set\_bounds** (const ColumnVector lb, const ColumnVector ub)

— : Bounds& **set\_lower\_bound** (int index, double low)  
— : Bounds& **set\_upper\_bound** (int index, double high)

— : Bounds& **set\_lower\_bounds** (double low)  
— : Bounds& **set\_upper\_bounds** (double high)

— : Bounds& **set\_lower\_bounds** (const ColumnVector lb)  
— : Bounds& **set\_upper\_bounds** (const ColumnVector ub)

— : ostream& **operator <<** (ostream &os, const Bounds &b)
